# Efficacy of Bromhexine hydrochloride combined with bronchoalveolar lavage under fiberoptic bronchoscopy in the treatment of mild to moderate community-acquired pneumonia

**DOI:** 10.12669/pjms.42.8.19507

**Published:** 2026-08

**Authors:** Feiyan Hu, Li Lin, Feijing Lv, Yanan Xu, Zhihui Dai

**Affiliations:** 1 Feiyan Hu Department of Respiratory and Critical Care Medicine, Yongkang First People’s Hospital, Yongkang, Zhejiang Province 321300, P.R. China; 2 Li Lin Department of Respiratory and Critical Care Medicine, Yongkang First People’s Hospital, Yongkang, Zhejiang Province 321300, P.R. China; 3 Feijing Lv Department of General Physician, Yongkang First People’s Hospital, Yongkang, Zhejiang Province 321300, P.R. China; 4 Yanan Xu Department of Respiratory and Critical Care Medicine, Yongkang First People’s Hospital, Yongkang, Zhejiang Province 321300, P.R. China; 5 Zhihui Dai Department of Respiratory and Critical Care Medicine, Yongkang First People’s Hospital, Yongkang, Zhejiang Province 321300, P.R. China

**Keywords:** Bronchoalveolar lavage, Bromhexine hydrochloride, Community-acquired pneumonia, Fiberoptic bronchoscopy

## Abstract

**Objective:**

To evaluate the clinical efficacy of bromhexine hydrochloride (BRH) combined with fiberoptic bronchoalveolar lavage (BAL) under fiberoptic bronchoscopy (FOB) in the treatment of patients with community-acquired pneumonia (CAP).

**Methodology:**

This retrospective cohort study included clinical data from 126 patients with mild-to-moderate CAP treated at Yongkang First People’s Hospital from March 2023 to March 2025. Of them, patients treated with BRH alone (BRH group, n=63) were matched 1:1 to a cohort treated with BRH combined with fiberoptic bronchoscopic BAL under FOB (BRH & BAL group, n=63). The primary outcome was clinical efficacy. Secondary outcomes included clinical symptoms, levels of inflammatory factors, and incidence of adverse reactions.

**Results:**

After treatment, the efficacy of the BRH & BAL group (95.2%) was significantly higher than that of the BRH group (84.1%) (P<0.05). Post-treatment duration of fever, time to cough relief, time to resolution of pulmonary rales, and hospital length of stay in the BRH & BAL group were all shorter than those in the BRH group (P<0.05). After treatment, the levels of IL-6, CRP, and PCT in both groups were considerably lower than pre-treatment and significantly lower in the BRH & BAL group than in the BRH group (P<0.05). There was no statistically significant difference in the incidence of adverse reactions between the two groups (P>0.05).

**Conclusion:**

BRH combined with BAL under FOB for the treatment of mild-to-moderate CAP is associated with improved therapeutic efficacy, alleviation of clinical symptoms, reduction of the inflammatory response, and good safety.

## INTRODUCTION

Community-acquired pneumonia (CAP), one of the most common infectious diseases in clinical practice, is associated with a high incidence, diverse clinical courses, and often slow recovery.^1^ Most patients with CAP present with mild-to-moderate severity. However, due to thick sputum, poor drainage, and persistent local inflammatory activation, some patients may experience prolonged fever, unrelieved cough, slow resolution of pulmonary rales, prolonged hospital stay, and even progression to severe CAP.^1^,^2^ Therefore, in addition to the conventional anti-infective and expectorant therapy, it is necessary to explore more effective and safe adjuvant treatment options.

Fiberoptic bronchoalveolar lavage (BAL) under fiberoptic bronchoscopy (FOB) is a minimally invasive, intuitive, and effective respiratory interventional technique that can directly clear sputum, pus plugs, inflammatory exudates, and pathogenic microorganisms in the airways and alveolar lumen, promoting the resolution of pulmonary lesions.^3^ At present, multiple guidelines and clinical studies have confirmed the efficacy of BAL under FOB in the treatment of severe pneumonia and have recommended it as an important life-saving and adjunctive therapeutic modality.^4^,^5^ However, its application value in mild to moderate CAP cases remains unclear, as most studies are conducted in patients with severe disease or those who fail conventional treatment.

In fact, patients with mild to moderate CAP also commonly experience airway secretion retention, difficulty expectorating sputum, and excessive local inflammatory responses.^6^ As a classic clinical mucolytic agent, bromhexine hydrochloride (BRH) can reduce sputum viscosity, promote ciliary beating, and improve airway clearance ability.^7^ However, relying solely on pharmacological expectoration has limited effects on clearing deep sputum plugs and inflammatory exudates in the alveoli.^8^ This study aimed to evaluate the efficacy of BRH combined with BAL under FOB in the treatment of mild-to-moderate CAP, aiming to provide evidence to optimize the treatment strategy for this group of patients.

## METHODOLOGY

Clinical records of patients with mild-to-moderate CAP treated between March 2023 and March 2025 at Yongkang First People’s Hospital were retrospectively analyzed. A total of 126 patients who received either BRH monotherapy or BRH combined with BAL under FOB were included. Among them, 63 patients who received BRH combined with BAL under FOB (BRH & BAL group) were matched 1:1 to a cohort treated with bromhexine hydrochloride (BRH group) using nearest-neighbor matching method, with matching criteria including gender, age, comorbidities, and disease severity.

### Ethical approval:

The ethics committee of our hospital approved the study with the number: 2026-LW-007-01-K, Date: March 13, 2026. Due to the retrospective nature of the study, the requirement for informed consent was waived by the Ethics Committee.

### Inclusion criteria:

- Met the diagnostic criteria for CAP and were diagnosed with mild to moderate CAP.^9^
- Pulmonary inflammation confirmed by chest CT or X-ray examination.
- Aged 18–80 years.

### Exclusion Criteria:

- Patients with severe pneumonia.
- Patients with severe dysfunction of vital organs such as the heart, liver, and kidneys.
- Patients with severe vital sign abnormalities (disturbance of consciousness, respiratory rate > 30 breaths/min, heart rate > 125 beats/min, systolic blood pressure < 90 mmHg, diastolic blood pressure < 60 mmHg, body temperature > 40°C, oxygen saturation < 92%).
- Significant findings on chest X-ray, such as multiple alveolar or bilateral lobe infiltrates, pleural effusion, and/or cavitation.
- Patients with autoimmune diseases or malignant tumors.
- Patients with severe complications such as respiratory failure and septic shock.

### Treatment methods:

All patients received conventional basic treatment, including anti-infection therapy (cephalosporins, quinolones, and other antibiotics selected based on initial etiological judgment, with subsequent adjustment according to microbiological test results and drug susceptibility testing), antitussive, antipyretic, and electrolyte balance maintenance interventions, as well as oxygen therapy (when necessary).

For patients in the BRH group, BRH injection was added to the conventional treatment due to inadequate response to conventional therapy alone. Dosage: 4-mg diluted in 100 mL of 0.9% sodium chloride injection, administered by intravenous infusion twice daily for 7–10 consecutive days.

Patients in the BRH & BAL group underwent BRH combined with BAL under FOB. The specific procedure was as follows: All patients fasted and restricted fluids for six hours prior to the procedure. Local anesthesia was administered (2% lidocaine aerosol inhalation). The patient was placed in a supine position, and a fiberoptic bronchoscope was inserted through the nasal or oral cavity and advanced slowly to the affected bronchial segments or subsegments. After observing the lesion site, lavage fluid (0.9% sodium chloride injection, approximately 37°C) was slowly instilled, 20–50 mL each time. After 30–60 seconds of retention, the lavage fluid was aspirated using a negative-pressure aspirator. This process was repeated 3–5 times until the aspirate was clear. Vital signs, including heart rate, blood pressure, and oxygen saturation, were closely monitored during lavage; if the patient experienced discomfort, the procedure was immediately stopped. BAL under FOB was performed 1–2 times per week.

### Primary outcome:

### Clinical efficacy:

The clinical efficacy criteria were formulated in accordance with the *Guidelines for the Clinical Application of Antimicrobial Agents*.

### Cure:

Complete resolution of clinical symptoms (fever, cough, sputum production, chest tightness, etc.), complete absorption of pulmonary inflammation on chest CT or X-ray, and normalization of blood routine and inflammatory factor levels.

### Marked effect:

Significant relief of clinical symptoms, absorption of most pulmonary inflammation (absorption area ≥70%) on chest CT or X-ray.

### Moderate:

***effect:*** Partial relief of clinical symptoms, partial absorption of pulmonary inflammation (absorption area 30%–69%) on chest CT or X-ray.

### Invalid:

No relief or even aggravation of clinical symptoms, no absorption or even enlargement of pulmonary inflammation on chest CT or X-ray.

Overall efficacy rate = (Number of cured cases + Number of cases with marked effect + Number of cases with moderate effect) / Total number of cases × 100%.^10^

Secondary Outcomes:

- Time to clinical symptom improvement, including duration of fever, time to cough relief, time to resolution of pulmonary rales, and hospital length of stay.
- Inflammatory factor levels, including interleukin-6 (IL-6), C-reactive protein (CRP), and procalcitonin (PCT) levels. Fasting venous blood samples (5 mL) were collected from patients before and after treatment. IL-6 and PCT levels were measured by enzyme-linked immunosorbent assay (ELISA); CRP levels were determined by immunoturbidimetry (Goldsite Aristo Ur, China). All kits were purchased from Goldsite Biotechnology (Shenzhen) Co., Ltd., and tests were performed strictly in accordance with the kit instructions.
- Adverse reactions, including gastrointestinal reactions such as nausea, vomiting, and diarrhea; neurological reactions such as dizziness and headache; and adverse reactions related to fiberoptic bronchoscopy (e.g., sore throat, hemoptysis, chest tightness). The incidence of adverse reactions was calculated.

### Statistical analysis:

Statistical analysis was performed using SPSS/PC statistical software (Version 27.0; IBM Corp, Armonk, NY, USA). Categorical data were expressed as n (%), and intergroup differences were analyzed using the chi-square test (e.g., gender, smoking status, comorbidities, disease severity, and microbial etiology). The normality of variables was assessed using visual methods (histograms and probability plots) and analytical methods (Kolmogorov-Smirnov/Shapiro-Wilk test). Measurement data following a normal distribution were expressed as mean ± standard deviation (SD). Differences between the two groups were analyzed using the independent-samples t-test, and within-group pre- and post-treatment comparisons were performed using the paired t-test. Non-normally distributed data were expressed as median and interquartile range. Intergroup comparisons were performed using the Mann-Whitney U test, and within-group pre- and post-treatment comparisons were performed using the Wilcoxon signed-rank test (e.g., IL-6, CRP, and PCT levels). Statistical significance was set at P<0.05.

## RESULTS

**Fig.1:**
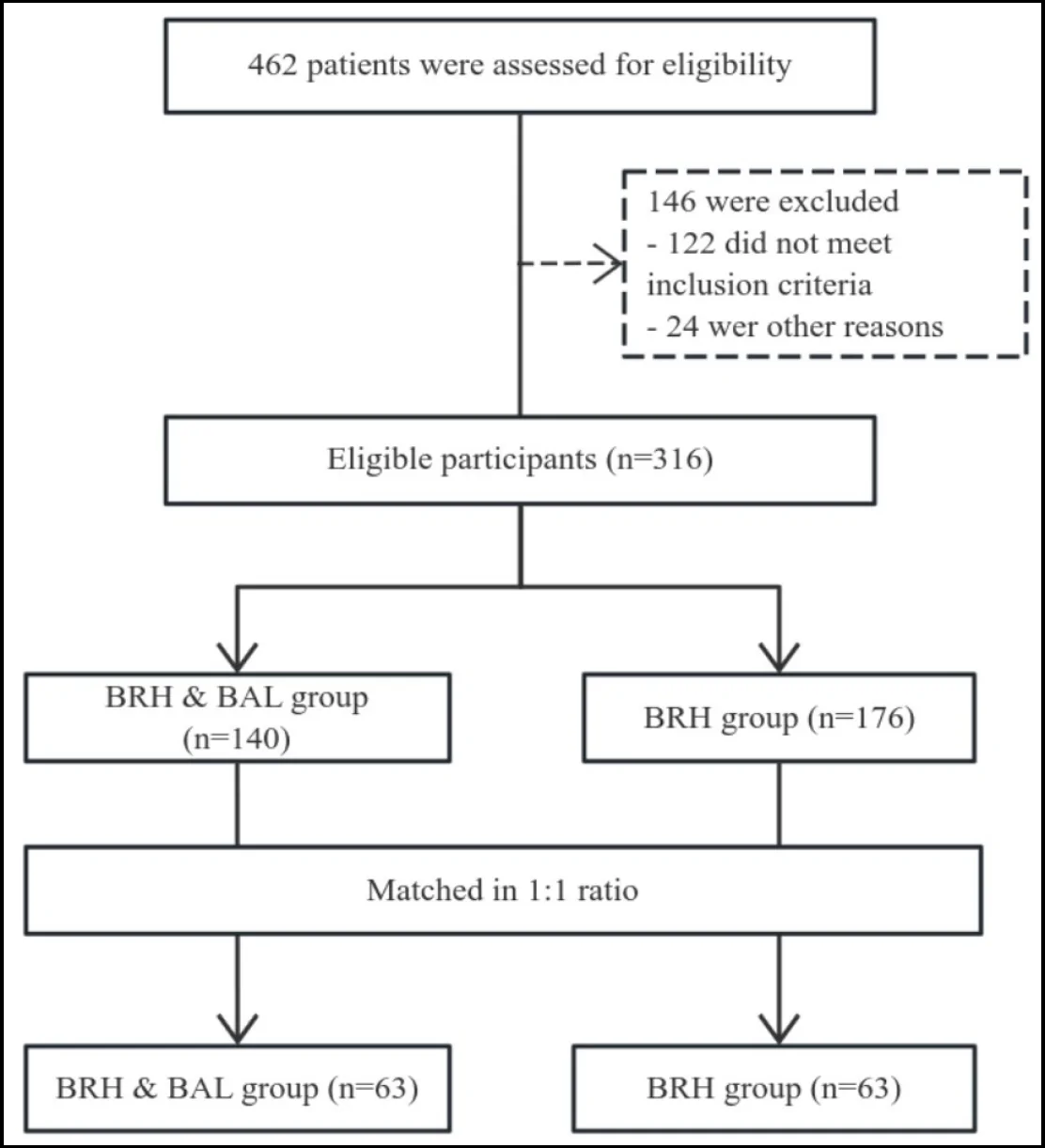
Flowchart of patient screening. BRH, bromhexine hydrochloride; BAL, bronchoalveolar lavage.

Clinical records of 462 patients with mild-to-moderate CAP were initially reviewed; 146 were excluded, and 316 met the inclusion criteria for this study (Fig.1). Of them, 176 patients received BRH monotherapy and 140 received BRH combined with BAL under FOB. Using gender, age, comorbidities, and disease severity as matching criteria, 1:1 matching was performed, resulting in 63 patients per group (Fig.1). There were no statistically significant differences in demographic characteristics between the two groups (P > 0.05; Table-I).

**Table-I:** Comparison of general characteristics between two groups of patients.

Characteristics	BRH & BAL group (n=63)	BRH group (n=63)	χ^2^/t	P
Gender, n(%)			0.797	0.372
Male	36 (57.1)	31 (49.2)		
Female	27 (42.9)	32 (50.8)		
Age (years)	55 (43, 62)	56 (46,63)	-	0.473
BMI (kg/m²)	23.2 (20.8, 25.4)	24.5 (21.3, 26.2)	-	0.123
Smoking, n(%)	31 (49.2)	27 (42.9)	0.511	0.475
Diabetes, n(%)	26 (41.3)	28 (44.4)	0.130	0.719
Hypertension n(%)	32 (50.8)	24 (38.1)	2.057	0.151
Severity, n(%)			0.519	0.471
Mild	29 (46.0)	25 (39.7)		
Moderate	34 (54.0)	38 (60.3)		
Pathogenic microbial sources, n(%)			2.543	0.280
Mycoplasma infection	20 (31.7)	16 (25.4)		
Bacterial infection	34 (54.0)	31 (49.2)		
Consider mixed infection	9 (14.3)	16 (25.4)		

-: Mann-Whitney U; BRH: bromhexine hydrochloride; BAL: bronchoalveolar lavage.

As shown in Table-II, the overall efficacy of the treatment was 95.2% in the BRH & BAL group, significantly higher than 84.1% in the BRH group (P<0.05). This indicates that the addition of BAL under FOB to BRH monotherapy can significantly improve therapeutic efficacy.

**Table-II:** Comparison of clinical effects between the two groups.

Group	Cure	Marked effect	Moderate effect	Invalid	Overall effective rate
BRH & BAL group (n=63)	16 (25.4)	39 (61.9)	5 (7.9)	3 (4.8)	60 (95.2)
BRH group (n=63)	10 (15.9)	35 (55.6)	8 (12.7)	10 (15.9)	53 (84.1)
*χ^2^*					4.203
*P*					0.04

BRH: bromhexine hydrochloride; BAL: bronchoalveolar lavage.

After treatment, the duration of fever, time to cough relief, time to resolution of pulmonary rales, and length of hospital stay were all significantly shorter in the BRH & BAL group than in the BRH group (P<0.05; Table-III). This indicates that the addition of BAL under FOB to BRH monotherapy can rapidly alleviate patients’ clinical symptoms.

**Table-III:** Clinical symptoms of the two groups after treatment, M (IQR).

Variables	BRH & BAL group (n=63)	BRH group (n=63)	P
Duration of fever (days)	3 (3, 5)	5 (4, 5)	0.005
Cough relief time (days)	4 (3, 5)	5 (4, 5)	<0.05
Lung rales decrease time (days)	4 (3, 5)	5 (4, 5)	0.001
Hospitalization time (days)	9 (7, 10)	9 (8, 10)	0.024

Before treatment, there were no statistically significant differences in IL-6, CRP, and PCT levels between the two groups (P > 0.05). As shown in Table-IV, post-treatment levels of IL-6, CRP, and PCT significantly decreased in both groups and were considerably lower in the BRH & BAL group than in the BRH group (P < 0.05). This indicates that adding BAL under FOB to BRH monotherapy can more effectively reduce the inflammatory response.

**Table-IV:** Comparison of IL-6, CRP, and PCT levels between the two groups, M (IQR).

Variables	BRH & BAL group (n=63)	BRH group (n=63)	P
***Before treatment***			
IL-6 (pg/mL)	31.47 (26.32, 38.94)	26.82 (22.95, 35.62)	0.135
CRP (mg/L)	24.22 (16.41, 35.09)	26.26 (17.48, 35.52)	0.169
PCT (ng/mL)	0.05 (0.03, 0.08)	0.05 (0.03, 0.09)	0.536
***After treatment***			
IL-6 (pg/mL)	17.45 (14.21, 23.12)*	22.14 (16.87, 26.03)*	0.003
CRP (mg/L)	16.73 (12.38, 22.84)*	20.28 (14.42, 25.64)*	0.042
PCT (ng/mL)	0.03 (0.02, 0.06)*	0.04 (0.03, 0.09)*	0.015

***Note:*** Compared with before treatment in the same group,

* P<0.05 BRH: bromhexine hydrochloride; BAL: bronchoalveolar lavage.

During the treatment period, there were two cases of gastrointestinal reactions, three cases of dizziness, and two cases of sore throat in the BRH & BAL group, with an adverse reaction rate of 11.1% (7/63). The BRH group reported two cases of gastrointestinal reactions and two cases of dizziness, with an adverse reaction rate of 6.4% (4/63). There was no statistically significant difference in the incidence of adverse reactions between the two groups (χ²=0.896, P=0.344) (Table-V). All adverse reactions were mild and resolved with symptomatic treatment, without affecting the treatment process.

**Table-V:** Comparison of adverse reactions between the two groups.

Adverse reactions	BRH & BAL group (n=63)	BRH group (n=63)	χ^2^	P
Gastrointestinal reactions	2 (3.2)	2 (3.2)		
Dizziness	3 (4.7)	2 (3.2)		
Sore throat	2 (3.2)	0 (0.0)		
Adverse reaction rate	7 (11.1)	4 (6.4)	0.896	0.344

## DISCUSSION

The study showed that BRH combined with BAL under FOB in the treatment of mild to moderate CAP is associated with improved overall efficacy, accelerated resolution of fever, cough, and pulmonary rales, shortened length of hospital stay, and a more effective reduction in inflammatory factors than BRH monotherapy. The combined treatment is not associated with increased adverse reactions, providing a reliable basis for the optimized treatment of mild-to-moderate CAP.

The overall effective rate of the BRH & BAL group was 95.2%, significantly higher than 84.1% in the BRH group, and the recovery time for symptoms and hospital stay was also shorter. Yang et al.^11^ and Fang et al.^12^ have confirmed the value of BRH combined with BAL under FOB in patients with severe CAP. The improved efficacy of the combined therapy is mainly due to BAL’s ability to directly and rapidly clear sputum, pus plugs, inflammatory exudates, and pathogens from the airways and alveoli, thereby reducing the infection load at the source.^13^ As BRH continuously reduces sputum viscosity and improves ciliary function, its combination with BAL under FOB provides a synergistic effect of local airway clearance and systemic expectoration.^11^–^14^ Similarly, this study observed the therapeutic effect of BRH combined with BAL under FOB in the treatment of mild to moderate CAP. The results showed that the addition of BAL under FOB to BRH could significantly improve therapeutic efficacy, accelerate symptom relief in patients with pneumonia, and shorten the course of the disease, especially for patients with thick sputum and expectoration disorders.^15^

In terms of inflammatory regulation, the results of this study demonstrated that the combined treatment was associated with more significant decreases in IL-6, CRP, and PCT levels. This is consistent with a recent study by Wu et al.^16^ on the treatment of elderly patients with severe pneumonia using acetylcysteine combined with BAL under FOB, which also showed that the addition of BAL under FOB to expectorants could significantly reduce the inflammatory response in patients. IL-6, CRP, and PCT are core indicators of infection severity, with higher levels indicating a more intense inflammatory response.^17^ BAL under FOB inhibits the release of inflammatory mediators from macrophages and epithelial cells by physically clearing pathogen-associated molecular patterns, thereby blocking the inflammatory cascade, whereas BAL under FOB also improves drainage and reduces local inflammatory stimulation.^18^,^19^ The combined therapy thus achieves a faster and stronger anti-inflammatory effect.

While current guidelines mostly recommend BAL under FOB for severe pneumonia,^18^–^20^ this study supports its appropriate use in eligible patients with mild to moderate CAP under the premise of strict indication selection, standardized operation, and safety control. Based on the results of this study, it is plausible that BRH combined with BAL under FOB treatment may serve as an effective early intervention that may block disease progression, promote accelerated recovery and shorten hospital stay, and improve the accuracy of etiological diagnosis with good safety. Indeed, as shown in this study, the combined approach was not associated with increased incidence of complications, and all observed adverse reactions were mild and transient, further providing basic clinical evidence supporting the feasibility of this regimen in patients with mild to moderate disease.

### Limitations:

First, this was a single-center retrospective analysis with a limited sample size, which may introduce selection biases. Moreover, the treatment allocation was not randomized, and selection bias may remain despite matching. Second, only the short-term efficacy of the patients was evaluated. Therefore, how the treatment affects patients’ long-term outcomes needs further study.

## CONCLUSION

BRH combined with BAL under FOB in the treatment of mild-to-moderate CAP is associated with improved clinical efficacy, alleviation of clinical symptoms, reduction of the inflammatory response, and good safety. When performed under standardized protocols and appropriate patient selection, this combined regimen appears safe and effective, offering a more proactive treatment option for patients with mild-to-moderate CAP.

### Authors’ contributions:

**FH** and **LL:** Literature search, study design and manuscript writing.

**FL, YX** and **ZD:** Data collection, data analysis and interpretation. Critical Review.

**FH** and **LL:** Manuscript revision and validation and is responsible for the integrity of the study.

All authors have read and approved the final manuscript.

### Recommendations:

Future multicenter randomized controlled studies can be conducted to perform stratified analyses by age and pathogens, extend follow-up time, and further verify long-term efficacy and safety.
